# Effects of reduced kinematic and social play experience on affective appraisal of human-rat play in rats

**DOI:** 10.1186/s12983-023-00512-0

**Published:** 2023-10-12

**Authors:** Quanxiao Liu, Tereza Ilčíková, Mariia Radchenko, Markéta Junková, Marek Špinka

**Affiliations:** Department of Ethology and Companion Animal Science, Faculty of Agrobiology, Food and Natural Resources, Czech University of Life Sciences, Prague, Czechia

**Keywords:** Affective appraisal, Animal development, Animal play, Kinematic play, Social play, Human-rat play, Norway rat, Ultrasonic vocalisation

## Abstract

**Background:**

Play is a common and developmentally important behaviour in young mammals. Specifically in Norway rats (*Rattus norvegicus*), reduced opportunity to engage in rough-and-tumble (RT) play has been associated with impaired development in social competence. However, RT play is a complex behaviour having both a kinematic aspect (i.e., performing complex 3D manoeuvres during play fights) and a social aspect (interacting with a playful partner). There has been little research so far on disentangling the two aspects in RT play, especially on how these two aspects affect the affective appraisal of the intense physical contact during play.

**Results:**

To examine the developmental effects of kinematic and social play reduction on affective appraisal in rats, we subjected male Long-Evans rats from 21 days old to RT play experience that was reduced either kinematically (through playing in a low ceiling environment) or socially (through playing with a less playful Fischer-344 rat). Starting at 35 days, we measured their production of positively (50-kHz) and negatively (22-kHz) valenced ultrasonic vocalisations (USVs) in a 2-min standardised human-rat play procedure that mimicked the playful sequences of nape contact, pinning, and belly stimulation (‘tickling’) for ten days. We hypothesised that the rats with kinematically or socially reduced play would perceive the ‘tickling’ less positively and thus emit positive ultrasonic vocalisations at lower rates compared to control rats with non-reduced play experience. Our results confirmed that each of the treatments reduced play differently: while the kinematic reduction abolished playful pinnings entirely, the social reduction decreased the pinnings and made play highly asymmetric. During the tickling procedure, rats mostly produced 50 kHz USV, indicating that they appraised the procedure as positive. There was a wide inter individual variance and high individual consistency in rats’ USV responses to ‘tickling’. Crucially, neither the kinematically nor the socially reduced play experience affected either type of USV production when rats were ‘tickled’.

**Conclusions:**

This finding indicates that the ability to appraise play-like interactions as positive remains unaffected even when the kinematic or the social aspect of play experience was substantially curtailed.

**Supplementary Information:**

The online version contains supplementary material available at 10.1186/s12983-023-00512-0.

## Background

Play is widespread among young mammals [1, 2], and the multifunction of play has fascinated researchers for decades [3, 4]. Pioneering studies have observed that playful juveniles have improved physical condition and survival [5, 6], better impulse control [7], better ability to cope with novel situations [8] and even improvements in reproduction [9]. In Norway rats (*Rattus norvegicus*), an established model species for animal play, experimentally deprived rough-and-tumble (RT) play opportunities reduce their social competence [10–12]. For instance, deprived rats tend to escalate encounters with strangers [13, 14]. In this study we built on the findings of these pioneering studies and examined developmental effects of two further aspects of animal play on social competence. First, as rat RT play is a complex package of kinematic (e.g. performing complex 3D manoeuvres) and social aspects (e.g. interacting with a playful partner), we attempted to disentangle these two aspects by reducing each of them separately in a different treatment. Second, we investigated whether the kinematically or socially reduced play experience compromised the rats’ ability to positively appraise the intense but playful physical contacts which may contribute to previously observed social incompetence.

Animal play is traditionally classified into three types: locomotor, object and social play. Locomotor play includes intense physical movements such as running, jumping, rotation, climbing, and swinging [15]; Object play refers to divertive manipulations with animate and inanimate objects like live and dead animals, plants, stones and sticks [16]; Social play encompasses behaviour like chasing, pouncing, wrestling, and mock fighting with other individuals [17]. These three categories of play provide the first insight into how nuanced the developmental effects of animal play are. Reduced locomotor play leads to decreased locomotor function and/or physicality in several species [5, 18, 19]. The experience of object play has been associated with differences in cognition [20, 21], and social play experience often has effects on social competence [22–24]. As useful as the traditional classification of play is, it still overlooks one crucial fact: the observed developmental effects of play are caused by play aspects that may overlap among play types.

The kinematic aspect of animal play, including movement, posture and coordination, is fundamental to all types of play. This is obvious for locomotor play, but also in most instances of social play where young animals perform sequences of body movements like chasing, grappling, pouncing, pinning and different kinds of rotations [25]. Similarly, in many instances of object play, animals perform fast movements while carrying an object, tearing it by fast head rotations or tossing it around [26]. Thus, the traditional classification can be enhanced by acknowledging that all play is kinematic, and that above this universal aspect, social play has the additional social aspect and object play has the additional object-manipulative aspect. In young mammals that play socially, it could be that specific developmental effects of play are either due to its kinematic or due to its social aspect. The potentially different developmental effects of different aspects of play have not been experimentally tested yet.

One often observed developmental effect of animal play is reduced social competence [10–12], where animals deprived of play tend to be aggressive and escalate encounters. One possible explanation for social incompetence is that these animals may not be able to appraise the encounters properly. Both kinematic and social aspects of play can have profound impact on developing the proper ability to appraise social encounters. For instance, physical exercises that resemble the kinematic aspect of play have been shown to stimulate neural development in the brain and spinal cord in humans [27] and this can too lead to improved cognitive abilities and coping with stress [28, 29] in developing animals. The kinematic aspect of play can also affect physiology, such as muscle growth [30, 31] and metabolism [32] via chronic exercise during play. As both cognition and physiology are fundamental to a wide range of behaviours [33, 34], young animals that had kinematically reduced play experience may develop a phenotype with a decreased ability to properly appraise affective situations. The social aspect of play requires young animals to perceive and process social signals to make social decisions. During play fights, for instance, successful turn-taking requires the animals to process social signals and match the reciprocity of their partners based on their age, sex, and dominance status [35–37]. Such social experiences can enhance communication and bonding skills in young animals and alter their development via hormonal regulation and stress responses, causing both immediate and long-lasting effects on their social behaviour [38–40], the lack of experience in the social aspect in play may lead to impoverished affective appraisal that could contribute to the social incompetence observed in previous studies. These studies examined the simultaneous deprivation of both kinematic and social aspects of play, leaving how these aspects individually contribute to the developmental effects of animal play unresolved. A better understanding of the interplay between these two aspects on affective appraisal could be achieved through studies that specifically disentangle their effects, offering a more nuanced picture of the developmental importance of animal play.

The Norway rat is a suitable laboratory species to disentangle the developmental effect of the kinematic and the social aspect of animal play on affective appraisal. Norway rats start to engage in RT play at about 15 days of age. Their play activity peaks at around 35 days and then gradually declines to a lower level at sexual maturity [41]. The RT play of Norway rats is highly kinematic and social. The ‘attacker’ initiates play by contacting the nape of its play partner (the ‘defender’). The ‘defender’ attempts to prevent the nape contact by either swerving or leaping away or by rotating to the supine position whereupon the ‘attacker’ pins the ‘defender’ to the ground before switching roles [42–44]. During RT play fights, rats emit a class of ultrasonic vocalisations (USVs) commonly denoted as’50-kHz calls’ [45] which are associated with positive effective states [46–48]. Rats also use another type of USV, commonly denoted as ’22-kHz calls’ to convey warnings and distress states [49, 50]. The emission rate of 50- and 22-kHz USVs can reflect how rats appraise their situation. A human-induced procedure (‘tickling’) that mimics the sequence of rat play fighting (i.e., nape contact, pinning, belly contact), also induces rats to emit positively valenced 50-kHz calls [51]. In rats, this tickling procedure has been repeatedly reported to receive other positive responses [52], further validating it as a reliable procedure to induce a positive state [53]. Although tickling is not exactly the same as conspecific play in rats [54, 55], in our study this procedure provides a controlled and standardised test that specifically assesses play appraisal by avoiding the complex social exchange that occurs during spontaneous rat-rat play. Therefore, tickling, combined with quantifiable measurements of both 50- and 22-kHz USVs, the direct vocal responses from rats, is specifically suitable for evaluating the affective appraisal of the rat.

To test the effects of both kinematic and social aspects of play on affective appraisal in rats, we subjected male Norway rats (Long Evans (LE) strain) to a reduced play environment starting at 21 days old. LE rats are widely used in rat play research, making them a reliable standard for our investigation. In the kinematically reduced environment, rats were allowed to play in a low-ceiling cage that restricted vertical movements such as leaping, rearing, pinning and rotating in play fights. This design aimed to reduce the occurrence of rat being pinned during intraspecies play as a ‘defender’, further contrasting this group’s play experience to the tickling procedure where the rats were put into the ‘defender’ position and constantly being pinned. In the socially reduced environment, the cage had a normal height, but the rats were paired with less social Fischer-344 (F-344) rats [13, 56, 57] rather than with LE counterparts. This group could experience the full kinematic experience of being pinned by a partner but with reduced social experience. Control rats played with LE partners in cages with a normal ceiling, with full kinematic and social experience of play. When the rats reached 35 days old, and after validating the RT play was indeed affected by both kinematically and socially reduced play treatments, we initiated daily tickling sessions for ten days and recorded the USVs during all tickling sessions. To assess the effect of having kinematically or socially reduced play experience on rats’ appraisal to the mimicked play fight (‘tickling’), we first compared both the positively valenced 50-kHz, as well as the warning 22-kHz USV production of the experimental groups to the control group. We then used a subset of data investigating whether rats emitted most USVs during belly and nape contact and if our treatments specifically affected USV emission during these contacts. We hypothesised that rats with reduced play experiences would display compromised affective appraisal, resulting in fewer 50-kHz USVs and more 22-kHz during tickling sessions than control rats, especially during ventral and dorsal contacts. Furthermore, we hypothesised that this effect may be different with reduced kinematic and reduced social aspects of play.

## Methods

### Subjects

Three batches of male LE rats (n_A_ = 17, n_B_ = 18, n_C_ = 18, at 21 ± 1 days old) were obtained from the Institute of Physiology of the Academy of Sciences of the Czech Republic on July 2021, October 2021 and March 2022. In each batch, the rats originated from four different litters. Additionally, with each batch, six F-344 rats were obtained from breeder VELAZ, s.r.o. at 21–28 days old. All rats were housed at one side of rat boxes (70 × 46 × 31 cm, brand: Ferplast Duna Multy Box, paper beddings brand: Pets Dream Paper Pure) separated in the middle by a wire mesh. Two rats in the same rat box had visual, auditory, olfactory and also limited tactile contact but the wire mesh barrier prevented physical interaction between the pair, such as play. Rats had *ad libitum* access to a standard laboratory rat diet and tap water. The rats were housed in an air-conditioned (22–24 °C) room (290 × 590 × 280 cm) with an artificial 14:10 L:D light cycle (lights on between 0800 and 2200 h).

### Kinematically and socially reduced play

In each batch, rats were randomly assigned to one of three groups: control, kinematically reduced and socially reduced. Rats from the same litter were equally assigned across the treatments whenever possible. Apart from the control group in the 1st batch (n_control_ = 5), all groups had 6 rats. All rats were allowed to play with their playmate (the rat from the same rat box) for one hour per work day (1000–1100 h, Monday to Friday) from 21 to 49 days old in a play cage (42 × 26 × 19.5 cm, plastic with wooden bedding). The playmates remained the same for the entirety of this experiment, however, the condition for playing differed between three treatments. Rats in the control group played with their LE playmate in an unaltered play cage. Rats in the kinematically reduced group also played with their LE playmate, however, their play arena was height restricted by a metal wire mesh ceiling (play arena height, week one: 3 cm, week two: 3.5 cm, week three: 4.0 cm, week four: 4.5 cm). This height restriction prevented vertical play elements such as leaping, rearing, pinning and rotating in rat play fights but rats could still touch, contact and crawl around each other. Rats in the socially reduced group played in an unaltered play arena but were paired with a F-344 rat to experience reduced social interactions during play. Each day, at 0955 h, we played back a sound stimulus (a loop of the Leporello Aria from Mozart’s Don Giovanni opera) for five minutes to announce the play session to the rats. At the end of the stimulus, we took all rats from their home cages, put them in play cages and left the room. One hour later, we came back and returned rats to their home cages. The 9th Rat RT play sessions were recorded for Batch A and C to assess how rat RT play was affected by our treatments. As play interactions mostly occurred during the first 10 min, we focused on the first 10 min and counted how many pinning (being pinned on the ground by its play mate) each individual rat received and delivered

### Recording of standardised human-rat play procedure

When rats reached 35 days old, we conducted a standardised human-rat play procedure (‘tickling’) to mimic the playful sequence of nape contact, supine pinning, and belly stimulation in rat RT play before their daily play sessions. This concurrent design can prevent rats from being entirely deprived of play or having same play experience during our repeated tickling sessions. The tickling was conducted once per day for the next ten work days between 0800 and 0930 h, resulting in ten sessions per rat for batches A and C. For batch B, due to practical and equipment constraints, there were only seven sessions per rat. Before the start of tickling sessions, a plastic tickling box (42 × 26 × 19.5 cm, wooden bedding was replaced after each rat) was placed on a table at a height of 93 cm at 100–300 cm distance to rats’ home boxes. A microphone (UltraSoundGate 116H, single-channel acquisition, and an CM16/CMPA condenser) was placed 28 cm above the tickling box to record the sound using a laptop running Avisoft-RECORDER software (version 4.2). Two cameras (Milesight) were placed in front and on the right side of the tickling box to record rats’ behaviour. Each rat was tickled for two minutes according to the procedure described in [58]. Each tickling session started by experimenters presenting a paper with the ID of the subject, date and USV recording number in front of one camera. Then the experimenter clapped in front of one camera (to synchronise audio and video recordings later), transferred the rat to the tickling box and hovered one hand in the middle of the tickling box. For the first 15 s, if the rat tried to sniff or touch the hand, the experimenter would slowly move the hand around the box until the rat stopped following it. In the next 15 s, the experimenter repeated the sequence mimicking RT play in rats, including tickling the nape three to four times with two fingers, flipping the rat onto its back, tickling its belly for three to four times and allowing the rat to straighten itself up onto its legs. The experimenter alternated between 15 s of hand hovering and 15 s of tickling for two minutes before ending the session and returning the rat to its home cage. The order of rats being tickled was fixed in each batch but rats from different treatments were randomly distributed along the tickling order. In each batch, the tickling was performed by a different person, instructed and trained to the procedure by one of the authors (MŠ).

### Automatic detection of 50- and 22-kHz USVs

A customised script (Additional file 1: Appendices 1 USV detection script) was created to automatically detect positively valenced 50-kHz USVs emitted during 2-min tickling sessions in R 4.1.2 [59]. Briefly (see Additional file 2: Appendices 2 Schematic of the automatic detection of 50- and 22-kHz USVs), for each audio recording, the script placed a sliding window of 0.5 s at the beginning of the recording. The audio within the window was bandpass filtered between 35 and 68 kHz and a Fourier transform (Hanning window, 1024 window length and 90% overlap) was applied to create a spectrogram with 1211 time windows (~ 0.45 ms each) and 136 frequency bands (~ 243 Hz each). The mean and the standard deviation of the amplitude values (linear scale, dBref = 2*10^e−5^) from frequency bands between 37.44 and 68 kHz were calculated for each of the 1211 time windows. The script then highlighted time windows with at least four frequency bands with amplitudes that exceeded the threshold equal to the mean plus 2.1 times the standard deviation. Highlighted time windows within 40 ms were grouped and if there were at least 16 highlighted time windows, the group was flagged as one detected 50-kHz USV. In the next step, the script removed all detected 50-kHz USVs shorter than 6.6 ms. The sliding window then advanced 0.5 s and the aforementioned process was repeated until the sliding window reached the end of the recording. To ensure the edges of the sidling window did not accidentally cut any USV in half, the detection process was repeated with the sliding window starting at 0.25 s into the recording. Detected USVs from both processes were combined and overlapping/connecting USVs were removed or merged. Finally, only 50-kHz USVs emitted during tickling sessions were selected, extracted and saved. The accuracy of this customised script was validated by having three experimenters manually screened ten random recordings for 50-kHz USVs, and the results were compared to the script's results. The accuracy of this customised script was above 99%. We used the same script to detect 22-kHz USVs but changed several parameters (bandpass filter between 18 to 26 kHz, plus 1.3 times of standard deviation of time window amplitude, require 2 flagged frequency bands, details please see Additional file 1: Appendices 1 USV detection script).

### Statistical analysis

We first tested whether rats in both kinematically and socially reduced play groups received fewer pinnings during RT play sessions. Because rats in the kinematically reduced group did not receive any pinnings, to prevent zero-inflation, we only included the control and the socially reduced group in this analysis. To this end, we created a generalised linear model (‘glm’ function in R package ‘lme4’) with the number of received pinnings as the response variable (Poisson distribution). The treatment (control, socially reduced) and batch (A, C) were the only fixed factors. We further investigated the asymmetry of the number of received and delivered pinnings for each rat. To this end, we first calculated the difference of delivered and received pinnings for each rats (P_d_ − P_r_). We then divided this difference by the sum of delivered and received pinnings to get an asymmetry score: Asymmetry score = (P_d_ − P_r_)/(P_d_ + P_r_). This asymmetry score ranged from − 1 (only received pinnings) to 1 (only delivered pinnings) and 0 indicated equal numbers of delivered and received pinnings. We created a linear model (‘lm’ function in R package ‘lme4’), with asymmetry score as the response variable and the treatment and batch as fixed factors.

Once validating that our treatments did affect rat RT play, we started to analyse how these treatments affected the emission rate of both 22- and 50-kHz USVs during tickling sessions. We tested how the kinematically or socially reduced play experience affected 50-kHz USV emission during entire tickling sessions. First, a correlation test was performed between the total duration and the total number of 50-kHz USVs in each session (n = 476). Since the total duration was highly correlated with the total number of 50-kHz USVs (r_s_ = 0.96, n = 476, *p* < 0.001), we used only the total number of 50-kHz USVs for further analyses. We created a linear mixed model (‘lmer’ function in R package ‘lme4’) with the total number of 50-kHz USVs per session as the response variable, treatment (control, kinematically reduced, socially reduced) and batch (A, B, C) as fixed factors, test day (1–10) and test order (1–18) as covariates, and rat ID and litter ID as random intercepts. We assumed a Gaussian distribution for response variable, as this distribution resulted in the best fitting model. In a top-down approach, we compared the full model to simpler models and found the full model to be the best model (Table 2a). We then conducted *post hoc* tests to further evaluate the differences between treatments using the best model (‘emmeans’ function in R package ‘emmeans’). To determine the repeatability of rat 50-kHz USV emission during tickling sessions, we estimated the repeatability for rat ID and litter ID (‘rpt’ function in R package ‘rptR’) using the best model. We also used the same method to test whether our treatments affected 22-kHz USV emission with the total number of 22-kHz USVs per session as the response variable. However, we assumed Poisson distribution for 22-kHz USVs because this distribution resulted in best fitting models.

We further examined whether rats emitted the 50-kHz USVs mostly when being ventral- or dorsal-contacted rather than when not being in contact with the human hand in a subset (batch C) of our tickling sessions. We first established time stamps of the beginning and the ending of every ventral contact (experimenters’ fingers contact the belly of the subject) and dorsal contact (experimenters’ fingers contact the nape of the subject) period. The rest of the recording was labelled as non-contact periods. In the corresponding audio recording, we counted how many detected 50-kHz USVs were emitted during each of these periods using the established time stamps. We then summed up the duration of all periods (in seconds) and the number of 50-kHz USVs for all ventral contact periods, all dorsal contact periods and all non-contact periods. After this, we calculated the emission rate of 50-kHz USVs per contact type by dividing the total number of 50-kHz USVs by the summed up duration of the corresponding period. Finally, we created a linear mixed model with the emission rate of 50-kHz USVs per session as the response variable. The interaction between contact types (ventral contact, dorsal contact, no contact), treatments and the summed up duration of contact phase (numeric) was the fixed factor. Test day was a covariate. Rat ID and litter ID were included as random intercepts. We followed the aforementioned model selection process (but included treatment as the fixed term) and in the best model we *post hoc* tested if rats from three groups differed in USV emission rate both when the rats were tickled or not tickled. Raw data can be found in Additional file 3: Apendices 3 Rat USV and play data.

## Results

**Fig. 1:**
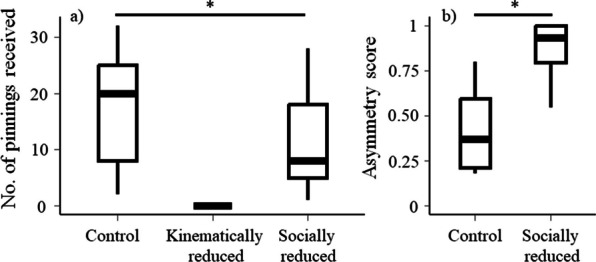
**a** Number of pinnings received during 10-min rat RT play and **b** Asymmetry score of paired rats in control and socially reduced groups. In **b**, high asymmetry score indicates the rat received fewer pinnings than delivered pinnings. In both figures, bars show the group median and the 1st and the 3rd quartile. Upper and lower whiskers extend from the bars to the largest/smallest value no further than 1.5 times of the distance between the first and third quartiles. The * sign indicates significant difference.

The experimental manipulation significantly reduced the pinnings received during rat RT play. Rats from the control group received more pinnings (mean [CIs] = 17.7 [11.8, 23.3]) than rats from both socially reduced (11.2 [6.8, 16.0]) and kinematically reduced group (did not receive any pinnings; Table 1a, Fig. 1a). The asymmetry score was also affected by our treatment (Table 1b, Fig. 1b). Rats from the socially reduced group received significantly fewer pinnings from their F-344 partners, compared to rats in the control group that were paired with LE rats.

**Table 1:** Summary of the statistical results obtained in the generalised linear model for received pinnings during rat RT play and the linear model for asymmetry score

Fixed effects	Estimate	Std. error	*p* Value
*(a) Number of received pinnings*			
**Intercept (control, Batch A)**	**3.20**	**0.08**	** < 0.001**
**Socially reduced**	** − 0.45**	**0.11**	** < 0.001**
**Batch C**	** − 0.82**	**0.12**	** < 0.001**
*(b) Asymmetry score*			
**Intercept (control, Batch A)**	**1.30**	**0.46**	**0.005**
**Socially reduced**	** − 1.33**	**0.48**	**0.006**
**Batch C**	** − 1.82**	**0.47**	** < 0.001**

Statistically significant effects are in bold

**Fig. 2:**
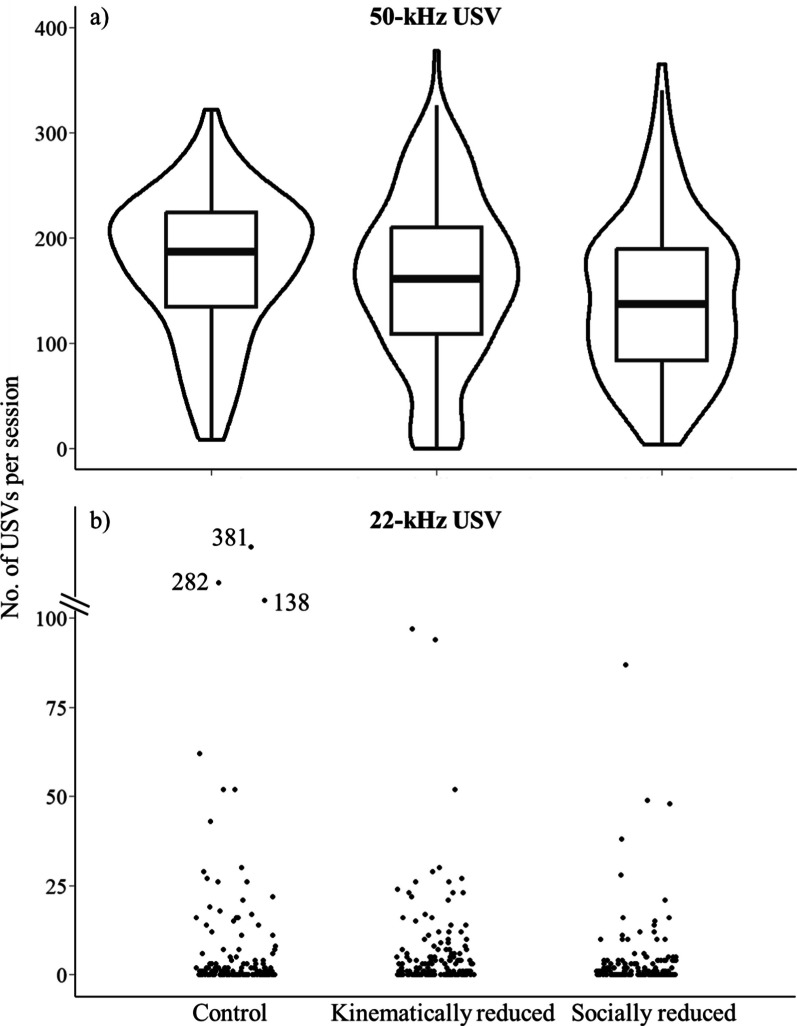
Effects of different play experience on the number of 50-kHz and 22-kHz USVs per tickling session. **a** 50-kHz USVs. The violin plot shows the distribution of number of 50-kHz USVs per tickling session. Bars show the group median and the 1st and the 3rd quartile. Upper and lower whiskers extend from the bars to the largest/smallest value no further than 1.5 times of the distance between the first and third quartiles. X-axis shows three treatment groups and y-axis shows the total number of 50-kHZ USVs per session. **b** 22-kHz USVs. Each dot represents one rat from one session. X-axis shows three treatment groups and y-axis shows the total number of 22-kHz USVs per session. Three extreme values above 100 on y-axis are labelled and displayed out of the scale

**Fig. 3:**
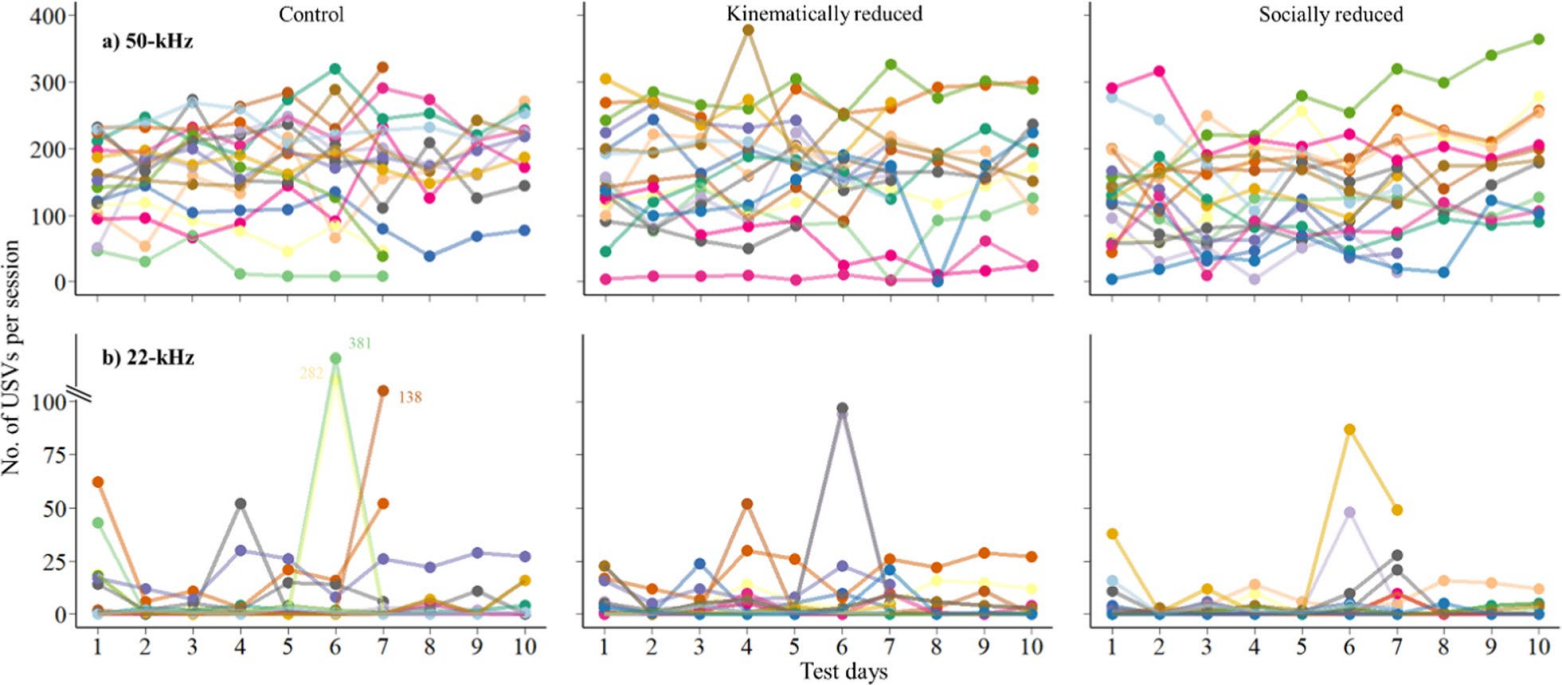
Individual repeatability of number of **a** 50-kHz and **b** 22-kHz USVs per tickling session. Each dot represents one tickling session and connected dots of the same colour are from the same individual. X-axis shows the consecutive test days and y-axis shows the number of 50-kHZ USVs per session. Each column shows one experimental condition

During tickling sessions, rats on average emitted 159 ± 75.7 50-kHz USVs per session. In the full (best) model, the treatment did not affect the 50-kHz USV emission (Fig. 2a, Tables 2a, 3a, 4a). In our *post hoc* analyses, control rats emitted more 50-kHz USVs (mean [CIs] = 176 [144, 208]) than rats with kinematically (167, [137, 198]) or socially (143 [111, 174]) reduced play experience but the differences were not significant. Rats increased 50-kHz USV emission at later testing days and rats tested later in the order had increased USV emission. The number of 50-kHz USVs per tickling session was highly repeatable in individual rats, regardless of treatments (Repeatability = 0.519 [0.306, 0.679], Fig. 3a) but not within litters (Repeatability = 0.137 [0, 0.365]).

**Table 2:** Top 5 models of the number of 50-kHz and 20-kHz USVs per tickling session from model section

	ΔAICc
*(a) Models for 50-kHz USVs*	
(1) Treatment + batch + days + order	0
(2) Treatment + batch + days	5.89
(3) Treatment + days + order	14.38
(4) Batch + days + order	14.61
(5) Treatment + batch + order	16.18
*(b) Models for 22-kHz USVs*	
(1) Batch + days	0
(2) Batch + days + order	1.94
(3) Treatment + batch + days	2.25
(4) Treatment + batch + days + order	4.22
(5) Days	15.89
*(c) Models for 50-kHz USV emission by contact periods*	
(1) Contact periods + treatment	0
(2) Contact periods + treatment + duration of the period	0.68
(3) Contact periods * treatment + duration of the period	3.54
(4) Contact periods * treatment	4.01
(5) Contact periods + treatment + Days	5.01

Rat IDs were included in all models as random intercepts. Litter IDs were included in (a) and (b) as random intercepts

**Table 3:** Factor level analysis of the fixed effects in the best models

Fixed effects	Sum of squares	F value	*p* Value
(a) 50-kHz USV best model			
Treatment	6454	1.72	0.19
**Days**	**32,336**	**17.32**	** < 0.001**
**Order**	**10,248**	**5.49**	**0.03**
Batch	2418	0.65	0.22
b) 22-kHz USV best model			
**Days**	**427.87**	**427.87**	** < 0.001**
**Batch**	**31.29**	**15.65**	** < 0.001**
c) USV emission by contact periods best model			
Treatment	0.62	1.14	0.35
**Contact periods**	**182.27**	**338.17**	** < 0.001**

Marginal and conditional r^2^ for the best 50-kHz USV model are 0.14 and 0.70, for the best 22-kHz USV model are 0.34 and 0.96, for the best USV emission by contact periods model are 0.40 and 0.72. Statistically significant effects are in bold

**Table 4:** Summary of the statistical results obtained in the linear mixed-effects model (best model)

Fixed effects	Estimate	Std. Error	*p* Value
*(a) 50-kHz USV best model*			
**Intercept (control, Batch A)**	**136.27**	**26.705**	** < 0.001**
Socially reduced	− 33.35	18.74	0.08
Kinetically reduced	− 8.59	18.77	0.64
**Days**	**3.09**	**0.742**	** < 0.001**
**Order**	**4.31**	**1.84**	**0.03**
Batch B	− 25.56	24.51	0.31
Batch C	− 23.58	26.67	0.40
*(b) 22-kHz USV best model*			
**Intercept (control, Batch A)**	− **0.72**	**0.33**	**0.03**
**Days**	**0.17**	**0.01**	** < 0.001**
**Batch B**	**2.13**	**0.45**	** < 0.001**
Batch C	− 0.01	0.45	0.99
*(c) USV emission by contact periods best model*			
**Intercept (control, no contact)**	**0.80**	**0.23**	**0.003**
Socially reduced	− 0.38	0.32	0.26
Kinematically reduced	− 0.46	0.32	0.18
**Ventral contact**	**1.24**	**0.05**	** < 0.001**
**Dorsal contact**	**1.22**	**0.05**	** < 0.001**

Statistically significant effects are in bold

The negative 22-kHz USVs were much less frequent compared to 50-kHz USVs, with rats emitting 23 ± 11.6 22-kHz USVs per session (Fig. 2b). Even this number was inflated by two sessions where two rats constantly emitted 22-kHz USVs, producing 321 and 282 vocalisations. Excluding these two sessions, the average emission of 22-kHz USVs is 12 ± 3.2 per session. Our treatment did not affect 22-kHz USV emission. Rats emitted more 22-kHZ USVs at later testing days (Tables 2b, 3b, 4b). 22-kHz USV emission was also consistent within individuals (Repeatability = 0.381 [0.193, 0.488], Fig. 3b) but not within litters (Repeatability = 0.02 [0, 0.048]). The emissions of 22-kHz and 50-kHz USVs were not correlated (r_s_ = − 0.02, n = 476, *p* = 0.62).

**Fig. 4:**
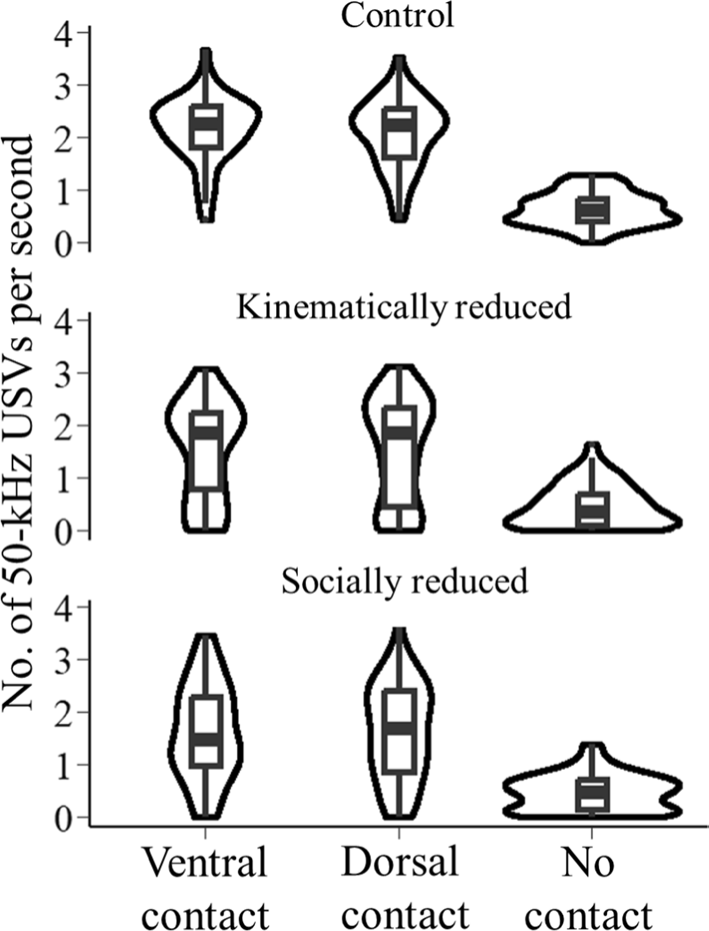
Emission rate of 50-kHz USVs during different contact periods. X-axis shows three periods and Y-axis shows the emission rate. The violin plots show the distribution of data. Bars show the group median and the 1st and the 3rd quartile. Upper and lower whiskers extend from the bars to the largest/smallest value no further than 1.5 times of the distance between the first and third quartiles. The treatment is labelled on top of the data

Rats from batch C emitted more 50 kHz USVs when being physically tickled (i.e., when in ventral or dorsal contact with the human hand) than during the non-contact periods of the tickling test (Fig. 4, Tables 2c, 3c, 4c). There were no significant differences between USV emission among the three treatments at any of contact periods (Fig. 4).

## Discussion

In our experiment, we succeeded in differentially reducing the kinematic and the social aspects of the rat RT play. Compared to rats in the control group, the rats in the kinematically reduced treatment were prevented from having any experience of being pinned by a playful partner as the low ceiling of the play arena did not allow one partner to be positioned above the other. The socially reduced treatment dramatically altered the reciprocity experience by making the play highly asymmetric with the experimental LE rats receiving just a fraction of the number of pinnings they delivered.

Despite the curtailed experience of play in two experimental groups, rats that experienced kinematically or socially reduced play did not differ in their production of either 50- or 22-kHz USVs during tickling sessions compared to control rats. Rats in all three treatments generally appraised being tickled as positive. On average, rats emitted 159 ± 75.7 50-kHz USVs per 2-min-long session and this number is on par with emissions from rats that had conventional play experience [51, 60–62]. In contrast, rats only emitted 23 ± 11.6 22-kHz USVs that are considered to be warning/aversive calls used in negatively valenced situations [50, 63, 64]. The high rate of 50-kHz USV combined with the low rate of 22-kHz USV indicates that rats reacted positively to tickling. Further analysis confirmed that in all three treatments, most of 50-kHz USVs were emitted when rats were being physically tickled on belly or nape by a human experimenter. Overall, the quantity and timing of USVs were very similar in three groups, indicating that the affective appraisal of playful ventral and dorsal contacts was not compromised by either of our treatments. Specifically, neither the deprivation of the kinematic aspect of play experience (i.e., having no experience of being pinned to the ground) nor the deprivation of the social aspect (i.e., not reciprocally delivering and receiving pinnings) altered the affective appraisal of the simulated play.

This is particularly interesting because play deprived rats were found to behave differently in rat-rat interactions [11, 13, 14] than rats with normal play experience. These studies in general found that play-deprived rats tend to escalate social encounters and exhibit more aggressive behaviour. A closer inspection (reviewed by [39]) reveals that the lack of reciprocity during play might be the key to atypical brain development and abnormal social behaviour in play-deprived rats. Our results suggest that the play-deprived rats, would still have developed the positive appraisal of play and likely want to engage in play as well. Thus, the previously observed aggressive behaviour and escalation may not be due to the lack of positive appraisal of play but rather may be a consequence of lacking expertise in coordinating and communicating with the stranger rat. This suggests that abilities to appraise and conduct social encounters are differently affected by play experience.

One possible explanation for why our treatment did not affect appraisal is that rats may require considerably less play experience to appraise the positive social situation of play, compared to what is needed to engage in it. In the present study, the tickled rats were in the ‘defender’ role and to appreciate tickling as a ‘defender’ may only require a minimal experience of RT play. In contrast, to engage in RT play, rats need to assess their partner and react accordingly, which would require considerable experience. It makes sense that the positive appraisal of play is an innate and rewarding mechanism [65–67] to motivate rats to seek and repeat play. So while the positive appraisal might be a largely innate mechanism that supports play, social competence may be a learned skill gained through play. Indeed, the positive appraisal of tickling was found to be robust even in rats that were submitted to the strong neuropsychological disturbance of a total 24 h sleep deprivation [68].

However, we do acknowledge that there are important differences between our study and previous studies. Our rats experienced reduced play since 25 days old and we concurrently tickled the rats when they were 35–45 days old. We specifically tested rats at this age range to maximise the potential effect. This is the developmental period of peak play motivation and, presumably, the positive appraisal of body contact is important to support the high play motivation in this period. As we stopped at day 45, we cannot tell whether potential developmental effects might merge later (e.g., early maturity around day 60), although this seems unlikely as no differences arose during the ages of peak play motivation in this study. However, it is possible that high play motivation may compensate for negative impacts of reduced play on positive appraisal. In any case, what our data clearly shows is that juvenile rats with no or highly asymmetric experience of pinning in RT play, immediately appraise being pinned and hand-tickled highly positively. Also, we tested the rats in a heterospecific play rather than in a conspecific way and USVs produced during tickling and rat RT play are not always aligned. For example, when playing with a devocalised partner, rats emitted more USVs at the pinning, rather than in the pinned position [69] while our rats emitted most USVs when being pinned (ventral contact) or held (dorsal contact) by a human experimenter. Previous study also suggested USVs production during tickling was not correlated with the number of pinning in RT play [70]. The lack of developmental effects on play appraisal from reduced kinematic and social play experience should also be tested in rat RT play in operant procedures that can evaluate how motivated the animals are to gain access to a peer to play with. Second, the effect of play deprivation was examined in juveniles in our study whereas many of the previous studies examined effects on adult play behaviour [12, 13, 71]. Therefore, further research is needed to investigate whether rats with especially kinematically reduced play experience as juveniles, will engage properly in conspecific social encounters later as adults.

Alternatively, it is possibility that the kinematically or socially reduced play increased the motivation to appraise tickling as more positive. Play deprivation is well-known to produce a rebound of play when the opportunity to accomplish this behaviour occurs [66, 72–74] and this heightened motivation to play is specifically caused by the previous lack of full physical interaction between animals [75]. It is possible that in the tickling session, the rats with kinematic or social play deprivation were more motivated to play, which may have compensated for their putatively compromised (or negatively shifted) play appraisal. Detailed analysis of rats’ behaviour during tickling sessions would provide insights in whether rats experienced kinematically and/or socially reduced play are more willing to play with human than control rats, e.g. whether rats increase their 50 kHz USVs in each non-contact period during one tickling session.

The production of 50-kHz USVs was positively correlated with the number of testing days, suggesting that rats gradually increased their affective appraisal of the tickling procedure. Although rats used in this study were extensively handled (at least daily during the transfer to the play cages and back), they were not previously exposed to the setup and the procedure of tickling. The increased affective appraisal was likely due to the familiarisation of our tickling procedure. Previous studies utilising similar tickling procedure mostly reported an increase in 50-kHz USV production in tickled rats [52], along with increased human approaching [76, 77], reduced handling reactivity [78–80] and reduced fear [81, 82]. However, because we tickled rats at age 35–45 days, it is also possible that these tickling sessions influenced the development of their general affective appraisal. But since we did not assess affective appraisal outside the tickling procedure, we do not have data to support this possibility.

We also found that rats tickled later on each day emitted more 50-kHz USVs. It is possible that the increase in USV emission was due to rats hearing the USVs produced by their conspecifics during tickling sessions. In our setup, rats in their home cages were situated between one to three meters from the tickling box and were not acoustically isolated so they could hear USVs produced by the tickled rats. 50-kHz USVs are important for social contact and communication among rats [46, 83]. Rats exposed to playbacks of 50-kHz USVs have a positive cognitive bias that more likely to treat ambiguous stimuli as positive, rather than as negative [48]. A similar cognitive bias could be induced among rats tickled later in our experiment so that they reacted more positively to being tickled than rats that had heard fewer 50-kHz USVs.

Tickling is increasingly recognised as a method to improve welfare of rats under laboratory conditions [52, 60, 80]. In accordance with consensus [51, 53, 55, 84, 85], our study revealed striking individual consistency and inter-individual variability in the emission of 50-kHz USVs, ranging from 14 to 280 USVs per individual per session. This indicates a large and stable difference in how rats appraise tickling and/or in how they communicate their appraisal vocally. When considering the use of tickling as a welfare improvement, it is important to be cautious about individual predispositions [54]. Future studies should investigate to what extent such individual differences originate from genetics (61) or other variables during early development, and whether and how this variability extends to affective appraising of other social interactions. Additionally, male and female rats differ in their social structure and endocrine system, indicating the effects of reduced play could affect sexes differently, follow-up studies should also investigate potential sex differences.

## Conclusions

In summary, our two experimental treatments differentially changed the play experience of rats. Nevertheless, neither kinematically nor socially reduced play experience altered the affective appraisal of human induced tickling in rats. Despite having reduced kinematic or social play experience of play, rats in treatment groups responded as positively to human tickling as the control rats. Our findings, combined with previous studies on the effects of deprived rat RT play, suggest that the ability to properly appraise and engage in play may be differently affected by the developmental effects of play. Being tickled puts the rat into the more passive ‘defender’ role in which the simpler and putatively more innate ability to appraise being contacted by the nape and belly is activated; whereas the more complex and proactive ability to reciprocally assess and respond to a play partner may require a full experience with play during early ontogeny. Further studies should investigate whether the lack of developmental effects from reduced kinematic and social play experience also holds in affective appraisal in rat-rat RT play. Additionally, studies using similar paradigm to specifically dissect different aspects of play experience are needed to explore the underlying mechanisms of rat RT play on development. Finally, our study highlights the significant individual variability in the appraisal of tickling, which adds a new layer to the welfare of laboratory animals.

### Supplementary Information

**Additional file 1. Appendices 1: **USV detection script.

**Additional file 2. Appendices 2: **Schematic of the automatic detection of 50- and 22-kHz USVs.

**Additional file 3. Appendices 3: **Rat USV and play data.

## Data Availability

All data generated or analysed during this study are included in this published article and its supplementary information files (Additional file 1: Appendices-1-USV-detection-script, Additional file 2: Appendices-2-Schematic of the automatic detection of 50- and 22-kHz USVs, Additional file 3: Apendices-3-Rat USV and play data).
