# Supplementary material for: Effects of reduced kinematic and social play experience on affective appraisal of human-rat play in rats
Source: Front Zool. 2023 Oct 12;20:34. doi: 10.1186/s12983-023-00512-0 (PMC10568924; doi:10.1186/s12983-023-00512-0)
Supplement: Supplementary file 1 — Additional file 1. Appendices 1: USV detection script. [file 12983_2023_512_MOESM1_ESM.docx]

Appendices 1 USV detection script:
Effects of reduced kinematic and social play experience on affective appraisal of human-rat play in rats

Quanxiao Liu*, Tereza Ilčíková, Mariia Radchenko, Markéta Junková, Marek Špinka

06-22-2023

## 50- and 22-kHz rat USV detection
### loading required R packages
library(multitaper)
library(seewave)
library(tuneR)
library(dplyr)
library(readr)
library(soundgen)
library(tidyr)
library(clValid)


## 50-kHz detection
### provide the path for the input .wav file containing 50-kHz USVs
path <- "please change this to your file path"

### create a function to detect 50-khZ USVs
#### threshold = time threshold to group detected time windows; margin = extension beyond the first and the last detection time window;
#### f = sampling rate; noise_scale = amplitude above how many times of the standard deviation + mean should be flagged;
#### detection_threshold = the minimal duration (based on the number of time windows) to be considered as one potential USV;
#### detection_threshold_2 = the minimal number of detected time window to group as one detected USV;
#### detection_res = the minimal number of frequency bands with amplitude above threshold to flag the time window;
#### from = starting time point of the sliding window; to = ending time point of the sliding window;
#### tag = tag to recognise which audio file this detection is performed on;
#### plot = plot the detection; plot_false = plot non-detection; store = store the time stamps of detected USVs
rat_vocal_detect_50khz <- function(wav, threshold = 30, margin = 2, f = 250000, noise_scale = 2.1, detection_threshold = 4, detection_threshold_2 = 4,
 from = 0, to = 0.5, tag = "", detect_res = 4,
 plot = TRUE, plot_false = TRUE, store = TRUE){
 ## load 0.5 seconds of the .wav file
 wav1 <- readWave(wav,
 from = from, to = to, units = 'seconds')
 ## extract spectrum from 35 to 68 khz and apply a hanning window with window lenght of 1024 and 90% overlap
 pas_ext <- ffilter(wav1, f = f, from = 35000, to = 68000, bandpass = TRUE,
 custom = NULL, wl = 1024, ovlp = 90, wn = "hanning", fftw = FALSE,
 rescale = FALSE, listen = FALSE, output = "Wave")
 ## extract amplitudes of this window
 sp_ext <- spectro(pas_ext,
 noisereduction = NULL, wl = 1024, ovlp = 90,
 scale = FALSE,
 fastdisp = TRUE, flim = c(35, 68),
 tlab = "", flab = "", alab = "", main = "", axisX = FALSE, axisY = FALSE,
 plot = FALSE)

 ## calculate the mean and the standard deviation of amplitudes for every time window
 time_scale <- length(sp_ext$time)
 sp_amp <- sp_ext$amp
 detect_points <- c()
 for (i in 1:time_scale){
 mean_sp <- mean(sp_amp[, i])
 sd_sp <- sd(sp_amp[, i])
 ## calculate the set threshold
 tr <- mean_sp + noise_scale*sd_sp
 t1 <- (sp_amp[c(11:136), i] > tr)
 ## flag frequency bands with amplitude value above the set threshold
 detect_points <- c(detect_points, length(t1[t1 == TRUE]))

 }
 ## flag time windows with enough frequency bands that have high amplitudes
 index_detect_points <- which(detect_points >= detect_res)

 if(length(index_detect_points) < 2){
 ## verbose if no detection
 print(paste('maybe no vocalisations, few detection: ', wav, tag))
 ## plot spectrogram of this 0.5 seconds of no detection (if allowed)
 if(plot_false){
 spectro(pas_ext, scale = FALSE,
 fastdisp = TRUE, flim = c(30, 85),
 tlab = "", flab = "", alab = "", main = "", axisX = FALSE, axisY = FALSE,
 noisereduction = 1)
 axis(1, at = c(0, 0.25), labels = c(as.character(from), as.character(from + 0.25)), col.axis = "black", las=1)
 }

 return()
 }else{
 start_seq <-c()
 end_seq <-c()
 ## if enough time windows are flagged, decide whether the time windows are one or multiple USVs
 i = 1
 start_seq <- c(start_seq, i)
 repeat{
 ### if there are more than 'detection_threshold_2' points within the set time threshold, treat these points within a single vocalisation
 f_temp <- index_detect_points[index_detect_points < (index_detect_points[i] + threshold) & index_detect_points >= index_detect_points[i]]
 if(length(f_temp) >= detection_threshold_2){
 i <- (i + length(f_temp) - 1)
 }else{
 end_seq <- c(end_seq, i)
 if(length(f_temp) != 1){
 i <- length(index_detect_points[index_detect_points <(index_detect_points[i] + threshold)])
 start_seq <- c(start_seq, i)
 }else{
 i <- i + 1
 start_seq <- c(start_seq, i)
 }
 }
 ### repeat until the last flagged time window
 if(i>= length(index_detect_points)){
 end_seq <- c(end_seq, i)
 break
 }
 }
 }

 ## remove grouped time windows that are too short to be vocalisations
 logic_temp <- (index_detect_points[end_seq] - index_detect_points[start_seq] >= detection_threshold)
 start_seq_cor <- start_seq[logic_temp]
 end_seq_cor <- end_seq[logic_temp]
 ## if there are still grouped time windows, get their time stamp with a margin
 if(length(start_seq_cor) > 0){
 s1_cor <- index_detect_points
 ## avoid time-stamp being too early
 s1_cor[start_seq_cor[index_detect_points[start_seq_cor] - margin < 0]] <- 0
 s1_cor[start_seq_cor[index_detect_points[start_seq_cor] - margin > 0]] <- index_detect_points[start_seq_cor[index_detect_points[start_seq_cor] - margin > 0]] - margin
 ## avoid time-stamp being too late
 s1_cor[end_seq_cor[index_detect_points[end_seq_cor] + margin > time_scale]] <- time_scale
 s1_cor[end_seq_cor[index_detect_points[end_seq_cor] + margin < time_scale]] <- index_detect_points[end_seq_cor[index_detect_points[end_seq_cor] + margin < time_scale]] + margin

 ## plot the results (if allowed)
 if(plot){

 spectro(pas_ext, scale = FALSE,
 fastdisp = TRUE, flim = c(30, 85),
 tlab = "", flab = "", alab = "", main = "", axisX = FALSE, axisY = FALSE,
 noisereduction = NULL)
 axis(1, at = c(0, 0.25), labels = c(as.character(from), as.character(from + 0.25)), col.axis = "black", las=1)
 segments(x0 = (s1_cor[start_seq_cor])*(to - from)/(time_scale), y0 = 80,
 x1 = (s1_cor[end_seq_cor])*(to - from)/(time_scale), y1 = 80,
 col = 'red', lwd = 5)
 print(paste('vocalisations: ', wav, tag))
 }
 ## store time stamps of USVs (if allowed)
 if(store){
 report_output <- data.frame(start = from + (s1_cor[start_seq_cor])*(to - from)/(time_scale),
 end = from + (s1_cor[end_seq_cor])*(to - from)/(time_scale))
 report_output
 }

 }else{
 print(paste('maybe no vocalisations: no cutting points', wav, tag))
 report_output <- data.frame(start = numeric(),
 end = numeric())
 report_output
 }
}


## set parameters to visualise results, please change these parameters accordingly

par(mfrow=c(4, 4))
par(mar=c(4,0,0,0))

## advance the sliding indow by 0.5 seconds until the end of the recording
### load recording
sound <- readWave(path,
 header=TRUE)
### calculate how many sliding windows are needed
dur_sec <- sound$samples/250000
t <- floor(dur_sec/0.5)
### create a dataframe to store time stamps
t1 <- data.frame(start = numeric(), end = numeric())

### analyse the entire recording
for(i in 1 : t){
 t2 <-rat_vocal_detect_65khz(path,
 f = 250000, threshold = 100, margin = 2, noise_scale = 2.1, detect_res = 4, detection_threshold = 16, detection_threshold_2 = 4,
 from = (i-1)*0.5, to = i*0.5, tag = as.character((i-1)*0.5), plot = TRUE, plot_false = TRUE)
 t2 <- t2
 t1 <<- rbind(t1, t2)

}

### connect USVs cut by the edge of the sliding window
i <- 1
repeat{
 if(i < nrow(t1)){
 if(t1$end[i] == t1$start[i + 1]){
 t1$end[i] <- t1$end[i+1]
 t1 <<- t1[-c(i+1),]
 i <- i -1
 }else{
 i <- i + 1
 }
 }else{
 break
 }
}

t_a <- t1


## repeat but with a sliding window starting at 0.25s
t1 <- data.frame(start = numeric(), end = numeric())
for(i in 1 : t){
 t2 <-rat_vocal_detect_60khz(path, threshold = 50, margin = 3, f = 250000, noise_scale = 1.5,
 from = (i-1)*0.5 + 0.25, to = i*0.5 + 0.25, tag = as.character((i-1)*0.5), plot = FALSE, plot_false = FALSE,
 detection_threshold = 1)
 t2 <- t2
 t1 <<- rbind(t1, t2)

}
### connect USVs cut by the edge of the sliding window
i <- 1
repeat{
 if(i < nrow(t1)){
 if(t1$end[i] == t1$start[i + 1]){
 t1$end[i] <- t1$end[i+1]
 t1 <<- t1[-c(i+1),]
 i <- i -1
 }else{
 i <- i + 1
 }
 }else{
 break
 }
}

t_b <- t1


## If USVs are overlapping from two analysis, merge them

t_total <- rbind(t_a, t_b)

t_total <- arrange(t_total, start)

cut_frame <- data.frame(start = numeric(),
 end = numeric())

if(nrow(t_total) > 0){
 if(nrow(cut_frame) > 0){
 for (j in 1:nrow(t_total)){
 ## check start
 ### if the new entry is later than the end -> new entry
 if(t_total[j, 1] >= tail(cut_frame, 1)[, 2]){
 cut_frame <- rbind(cut_frame, t_total[j, ])
 }else{
 cut_frame[nrow(cut_frame), 2] <- t_total[j, 2]
 }
 }
 }else{
 cut_frame <- rbind(cut_frame, t_total[1, ])
 }
}


## batch analysis using all aforementioned functions
### the path of a folder containing multiple .wav recordings of USVs
path_folder <- "change this to your own folder path"
file_list <- list.files(path_folder, pattern = '.wav', full.names = TRUE)


### batch analysis
for (i in 1: length(file_list)){

 sound <- readWave(file_list[i],
 header = TRUE)
 dur_sec <- sound$samples/250000
 t <- floor(dur_sec/0.5)

 ### the last thing to run
 t1 <- data.frame(start = numeric(), end = numeric())

 for(j in 1 : t){
 t2 <-rat_vocal_detect_65khz(file_list[i],
 f = 250000, threshold = 100, margin = 2, noise_scale = 2.1, detect_res = 4, detection_threshold = 16, detection_threshold_2 = 4,
 from = (j-1)*0.5, to = j*0.5, tag = as.character((j-1)*0.5), plot = FALSE, plot_false = FALSE, store = TRUE)
 t1 <<- rbind(t1, t2)

 }


 l <- 1
 repeat{
 if(l < nrow(t1)){
 if(t1$end[l] == t1$start[l + 1]){
 t1$end[l] <- t1$end[l+1]
 t1 <<- t1[-c(l+1),]
 }else{
 l <- l + 1
 }
 }else{
 break
 }
 }

 t_a <- t1

 t1 <- data.frame(start = numeric(), end = numeric())
 for(j in 1 : (t-1)){
 t2 <-rat_vocal_detect_65khz(file_list[i],
 f = 250000, threshold = 100, margin = 2, noise_scale = 2.1, detect_res = 4, detection_threshold = 16, detection_threshold_2 = 4,
 from = (j-1)*0.5+0.25, to = j*0.5+0.25, tag = as.character((j-1)*0.5+0.25), plot = FALSE, plot_false = FALSE, store = TRUE)
 t1 <<- rbind(t1, t2)

 }

 l <- 1
 repeat{
 if(l < nrow(t1)){
 if(t1$end[l] == t1$start[l + 1]){
 t1$end[l] <- t1$end[l+1]
 t1 <<- t1[-c(l+1),]
 }else{
 l <- l + 1
 }
 }else{
 break
 }
 }

 t_b <- t1

 t_total <- rbind(t_a, t_b)

 t_total <- arrange(t_total, start)

 cut_frame <- data.frame(start = numeric(),
 end = numeric())

 if(nrow(t_total) > 0){
 cut_frame <- rbind(cut_frame, t_total[1, ])
 for (k in 1:nrow(t_total)){
 ## check start
 ### if the new entry is later than the end -> new entry
 if(t_total[k, 1] >= tail(cut_frame, 1)[, 2]){
 cut_frame <- rbind(cut_frame, t_total[k, ])
 }else{
 cut_frame[nrow(cut_frame), 2] <- t_total[k, 2]
 }
 }
 }


 ### set the output folder for detection result
 setwd("change this to your own folder")

 save_path <- strsplit(file_list[i], "/")[[1]][5]
 save_path <- strsplit(save_path, ".wav")[[1]][1]
 save_path <- paste(save_path, ".csv", sep = "")
 write.csv(cut_frame, save_path)

}

### combine segmented detection with minimal interval of 20 ms
path <- "the folder contain all detection results"
file_list <- list.files(path, pattern = '.csv', full.names = TRUE)
file_list_short <- list.files(path, pattern = '.csv', full.names = FALSE)
### set the output folder for refined detection results
setwd("E:/Rat/B_detect_P")

for (i in 1:length(file_list)){
 read_in <- read_csv(file_list[i])
 read_in <- data.frame(read_in)
 line_n <- nrow(read_in)
 output_name <- paste("C_", file_list_short[i], sep = "")
 if(line_n >0){
 j <- 1
 repeat{
 if(j < line_n){
 if(read_in[j+1, 2] - read_in[j, 3] < 0.02){
 read_in[j, 3] <- read_in[j+1, 3]
 read_in <<- read_in[-c(j+1),]
 line_n <- nrow(read_in)
 }else{
 j <- j + 1
 }
 }else{
 break
 }
 }
 }
## delete short vocals < 10ms
 if(line_n >0){
 j <- 1
 repeat{
 if(j <= line_n){
 if(read_in[j, 3] - read_in[j, 2] < 0.01){
 read_in <<- read_in[-c(j),]
 line_n <- nrow(read_in)
 }else{
 j <- j + 1
 }
 }else{
 break
 }
 }
 }

 write.csv(read_in, output_name)
}


## 22khz detection
### create a function to detect 22khz USVs (same as 50-kHz detection but with different settings)
rat_vocal_detect_22khz <- function(wav, threshold = 30, margin = 2, f = 250000, noise_scale = 1.3, detection_threshold = 2, detection_threshold_2 = 2,
 from = 0, to = 0.5, tag = "", detect_res = 4,
 plot = TRUE, plot_false = TRUE, store = TRUE){
 wav1 <- readWave(wav,
 from = from, to = to, units = 'seconds')
 pas_ext <- ffilter(wav1, f = f, from = 18000, to = 26000, bandpass = TRUE,
 custom = NULL, wl = 1024, ovlp = 90, wn = "hanning", fftw = FALSE,
 rescale = FALSE, listen = FALSE, output = "Wave")
 sp_ext <- spectro(pas_ext,
 noisereduction = NULL, wl = 1024, ovlp = 90,
 scale = FALSE,
 fastdisp = TRUE, flim = c(18, 30),
 tlab = "", flab = "", alab = "", main = "", axisX = FALSE, axisY = FALSE,
 plot = FALSE)

 time_scale <- length(sp_ext$time)
 sp_amp <- sp_ext$amp
 detect_points <- c()
 for (i in 1:time_scale){
 mean_sp <- mean(sp_amp[, i])
 sd_sp <- sd(sp_amp[, i])
 tr <- mean_sp + noise_scale*sd_sp
 t1 <- (sp_amp[c(1:50), i] > tr)
 detect_points <- c(detect_points, length(t1[t1 == TRUE]))

 }

 index_detect_points <- which(detect_points >= detect_res)

 if(length(index_detect_points) < 2){
 print(paste('maybe no vocalisations, few detection: ', wav, tag))
 if(plot_false){
 spectro(pas_ext, scale = FALSE,
 fastdisp = TRUE, flim = c(18, 30),
 tlab = "", flab = "", alab = "", main = "", axisX = FALSE, axisY = FALSE,
 noisereduction = 1)
 axis(1, at = c(0, 0.25), labels = c(as.character(from), as.character(from + 0.25)), col.axis = "black", las=1)
 }

 return()
 }else{
 start_seq <-c()
 end_seq <-c()

 i = 1
 start_seq <- c(start_seq, i)
 repeat{
 f_temp <- index_detect_points[index_detect_points < (index_detect_points[i] + threshold) & index_detect_points >= index_detect_points[i]]
 if(length(f_temp) >= detection_threshold_2){
 i <- (i + length(f_temp) - 1)
 }else{
 end_seq <- c(end_seq, i)
 if(length(f_temp) != 1){
 i <- length(index_detect_points[index_detect_points <(index_detect_points[i] + threshold)])
 start_seq <- c(start_seq, i)
 }else{
 i <- i + 1
 start_seq <- c(start_seq, i)
 }
 }
 if(i>= length(index_detect_points)){
 end_seq <- c(end_seq, i)
 break
 }
 }
 }

 logic_temp <- (index_detect_points[end_seq] - index_detect_points[start_seq] >= detection_threshold)
 start_seq_cor <- start_seq[logic_temp]
 end_seq_cor <- end_seq[logic_temp]
 if(length(start_seq_cor) > 0){
 s1_cor <- index_detect_points
 s1_cor[start_seq_cor[index_detect_points[start_seq_cor] - margin < 0]] <- 0
 s1_cor[start_seq_cor[index_detect_points[start_seq_cor] - margin > 0]] <- index_detect_points[start_seq_cor[index_detect_points[start_seq_cor] - margin > 0]] - margin
 s1_cor[end_seq_cor[index_detect_points[end_seq_cor] + margin > time_scale]] <- time_scale
 s1_cor[end_seq_cor[index_detect_points[end_seq_cor] + margin < time_scale]] <- index_detect_points[end_seq_cor[index_detect_points[end_seq_cor] + margin < time_scale]] + margin

 if(plot){


 spectro(pas_ext, scale = FALSE,
 fastdisp = TRUE, flim = c(18, 30),
 tlab = "", flab = "", alab = "", main = "", axisX = FALSE, axisY = FALSE,
 noisereduction = NULL)
 axis(1, at = c(0, 0.25), labels = c(as.character(from), as.character(from + 0.25)), col.axis = "black", las=1)
 segments(x0 = (s1_cor[start_seq_cor])*(to - from)/(time_scale), y0 = 28,
 x1 = (s1_cor[end_seq_cor])*(to - from)/(time_scale), y1 = 28,
 col = 'red', lwd = 5)
 print(paste('vocalisations: ', wav, tag))
 }

 if(store){
 report_output <- data.frame(start = from + (s1_cor[start_seq_cor])*(to - from)/(time_scale),
 end = from + (s1_cor[end_seq_cor])*(to - from)/(time_scale))
 report_output
 }


 }else{
 print(paste('maybe no vocalisations: no cutting points', wav, tag))
 report_output <- data.frame(start = numeric(),
 end = numeric())
 report_output
 }
}


par(mfrow=c(4, 4))
par(mar=c(4,0,0,0))
sound <- readWave(path,
 header=TRUE)
dur_sec <- sound$samples/250000
t <- floor(dur_sec/0.5)
t1 <- data.frame(start = numeric(), end = numeric())

### settings for 22-khz detection
for(i in 1 : t){
 t2 <-rat_vocal_detect_22khz(path, f = 250000,
 threshold = 60, margin = 2, noise_scale = 1.3, detect_res = 2, detection_threshold = 2, detection_threshold_2 = 2,
 from = (i-1)*0.5, to = i*0.5, tag = as.character((i-1)*0.5), plot = TRUE, plot_false = FALSE)
 t2 <- t2
 t1 <<- rbind(t1, t2)

}


path_folder <- "change this to your own folder"
file_list <- list.files(path_folder, pattern = '.wav', full.names = TRUE)

for (i in 1: length(file_list)){

 sound <- readWave(file_list[i],
 header = TRUE)
 dur_sec <- sound$samples/250000
 t <- floor(dur_sec/0.5)

 t1 <- data.frame(start = numeric(), end = numeric())

 for(j in 1 : t){
 t2 <- rat_vocal_detect_22khz(file_list[i], f = 250000,
 threshold = 60, margin = 2, noise_scale = 1.3, detect_res = 2, detection_threshold = 2, detection_threshold_2 = 2,
 from = (j-1)*0.5, to = j*0.5, tag = as.character((j-1)*0.5), plot = FALSE, plot_false = FALSE)
 t1 <<- rbind(t1, t2)

 }

 l <- 1
 repeat{
 if(l < nrow(t1)){
 if(t1$end[l] == t1$start[l + 1]){
 t1$end[l] <- t1$end[l+1]
 t1 <<- t1[-c(l+1),]
 }else{
 l <- l + 1
 }
 }else{
 break
 }
 }

 t_a <- t1

 t1 <- data.frame(start = numeric(), end = numeric())
 for(j in 1 : (t-1)){
 t2 <-rat_vocal_detect_22khz(file_list[i], f = 250000,
 threshold = 60, margin = 2, noise_scale = 1.3, detect_res = 2, detection_threshold = 2, detection_threshold_2 = 2,
 from = (j-1)*0.5+0.25, to = j*0.5+0.25, tag = as.character((j-1)*0.5+0.25), plot = FALSE, plot_false = FALSE)
 t1 <<- rbind(t1, t2)

 }

 l <- 1
 repeat{
 if(l < nrow(t1)){
 if(t1$end[l] == t1$start[l + 1]){
 t1$end[l] <- t1$end[l+1]
 t1 <<- t1[-c(l+1),]
 }else{
 l <- l + 1
 }
 }else{
 break
 }
 }

 t_b <- t1

 t_total <- rbind(t_a, t_b)

 t_total <- arrange(t_total, start)

 cut_frame <- data.frame(start = numeric(),
 end = numeric())

 if(nrow(t_total) > 0){
 cut_frame <- rbind(cut_frame, t_total[1, ])
 for (k in 1:nrow(t_total)){
 if(t_total[k, 1] >= tail(cut_frame, 1)[, 2]){
 cut_frame <- rbind(cut_frame, t_total[k, ])
 }else{
 cut_frame[nrow(cut_frame), 2] <- t_total[k, 2]
 }
 }
 }


 setwd("your own folder")

 save_path <- strsplit(file_list[i], "/")[[1]][5]
 save_path <- strsplit(save_path, ".wav")[[1]][1]
 save_path <- paste(save_path, ".csv", sep = "")
 write.csv(cut_frame, save_path)


}

### combine segmented detection with minimal interval of 20 ms
path <- "your own folder"
file_list <- list.files(path, pattern = '.csv', full.names = TRUE)
file_list_short <- list.files(path, pattern = '.csv', full.names = FALSE)

setwd("your own folder")

for (i in 1:length(file_list)){
 read_in <- read_csv(file_list[i])
 read_in <- data.frame(read_in)
 line_n <- nrow(read_in)
 output_name <- paste("C_", file_list_short[i], sep = "")
 if(line_n >0){
 j <- 1
 repeat{
 if(j < line_n){
 if(read_in[j+1, 2] - read_in[j, 3] < 0.02){
 read_in[j, 3] <- read_in[j+1, 3]
 read_in <<- read_in[-c(j+1),]
 line_n <- nrow(read_in)
 }else{
 j <- j + 1
 }
 }else{
 break
 }
 }
 }
 if(line_n >0){
 j <- 1
 repeat{
 if(j <= line_n){
 if(read_in[j, 3] - read_in[j, 2] < 0.01){
 read_in <<- read_in[-c(j),]
 line_n <- nrow(read_in)
 }else{
 j <- j + 1
 }
 }else{
 break
 }
 }
 }


 write.csv(read_in, output_name)
}
