# Supplementary material for: Effects of reduced kinematic and social play experience on affective appraisal of human-rat play in rats
Source: Front Zool. 2023 Oct 12;20:34. doi: 10.1186/s12983-023-00512-0 (PMC10568924; doi:10.1186/s12983-023-00512-0)
Supplement: Supplementary file 2 — Additional file 2. Appendices 2: Schematic of the automatic detection of 50- and 22-kHz USVs. [file 12983_2023_512_MOESM2_ESM.docx]

Appendices 2

**Effects of reduced kinematic and social play experience on affective appraisal of human-rat play in rats**

Quanxiao Liu*, Tereza Ilčíková, Mariia Radchenko, Markéta Junková, Marek Špinka

**Schematic of the automatic detection of 50- and 22-kHz USVs**

Step 1. Place a sliding window of 0.5 seconds (red square in the figure) at the start of audio recording. For 50-kHz USV detection, apply a bandpass filter 35 to 68 kHz (For 22-kHz USVs, 18 to 22 kHz) within this window and apply Fourier transformation with a Hanning window with window length of 1024 and 90% overlap to create a spectrogram.


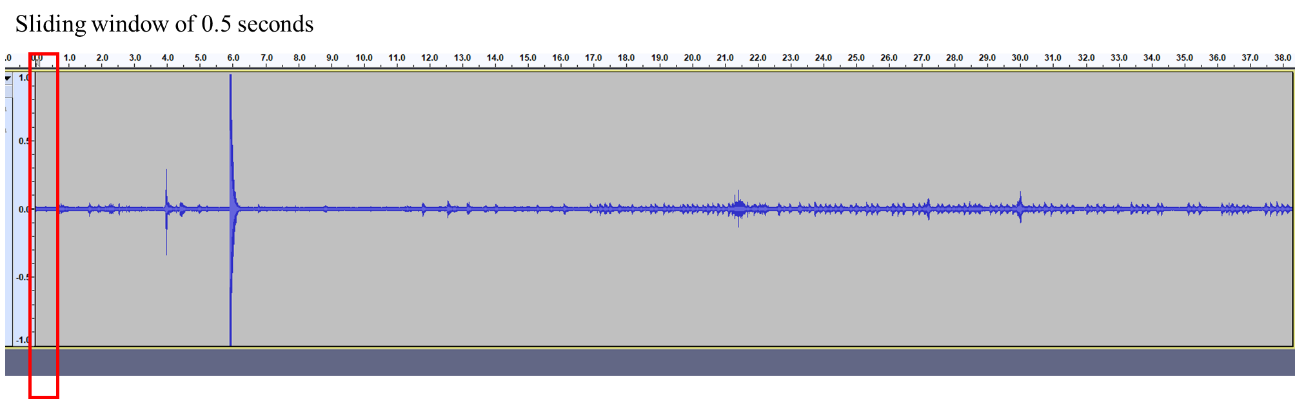


Step 2. Within this spectrogram, calculate means and standard deviations for amplitudes of all frequency bands on each time window. Add the mean with the multiplied setting (50-kHz USV, 2.1; 22-kHz USV 1.3) standard deviation to create a detection threshold. Flag all frequency bands with a amplitude above the set threshold.


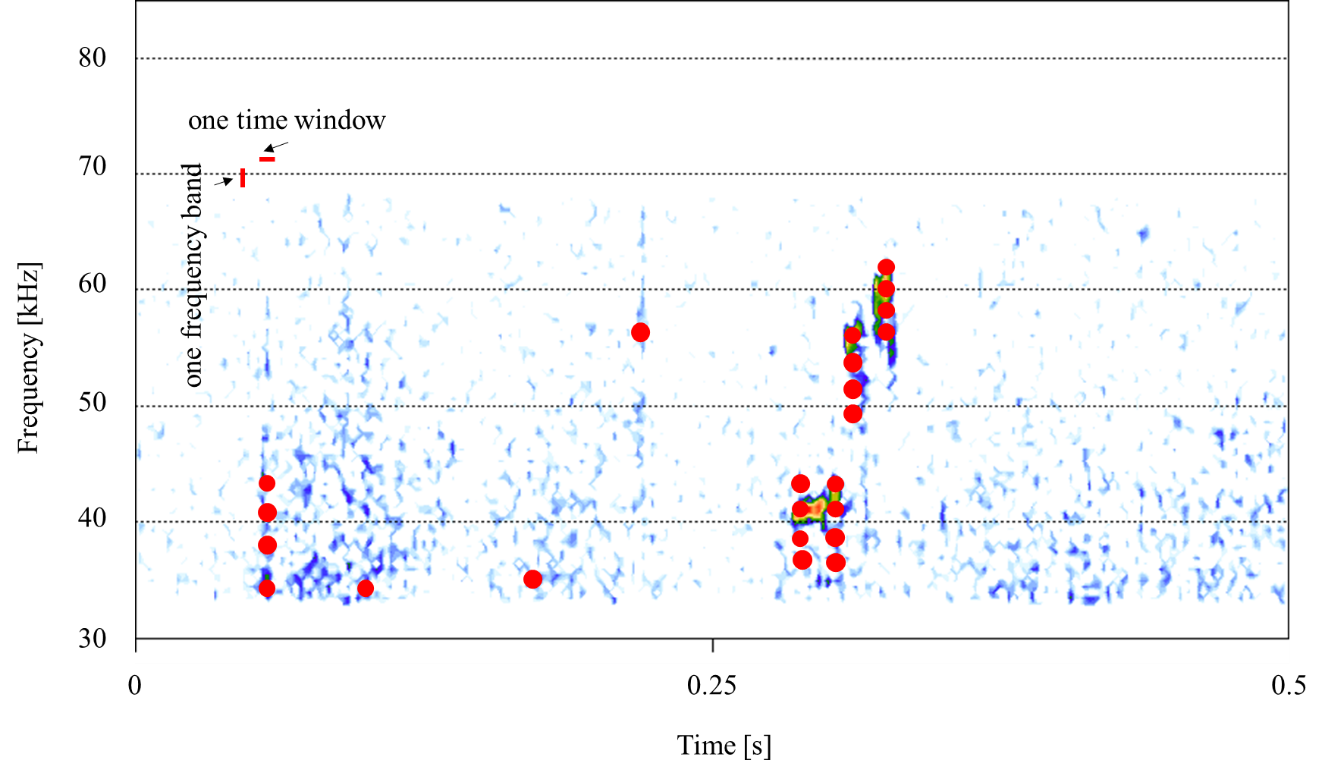


Step 3. Group nearby (< 40ms) time windows with more flagged frequency bands than the set value (50-kHz, 4; 22-kHz, 2;). Highlight this group as one potential USV.


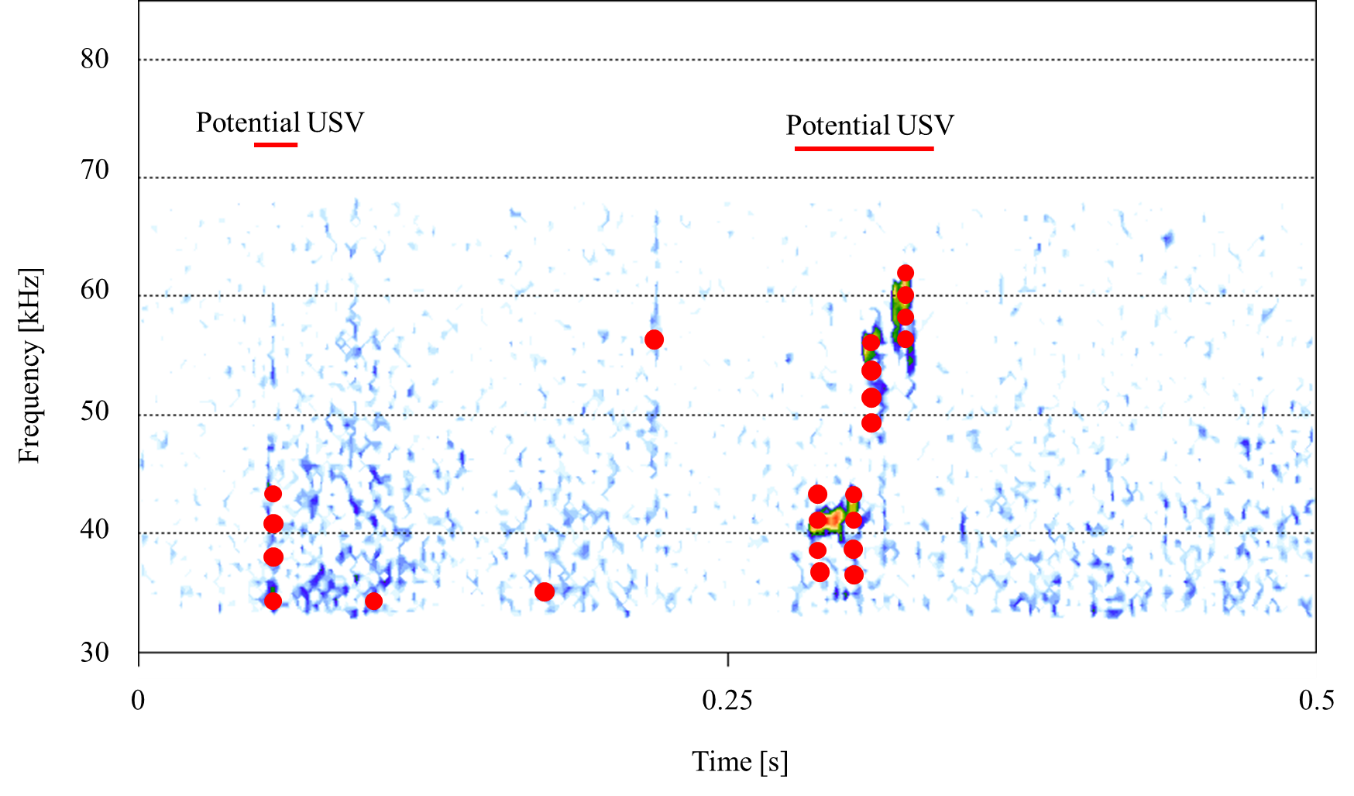


Step 4. Remove all highlighted potential USVs that are too short (< 6.6 ms). Report the remaining potential USVs as true detections of USV.


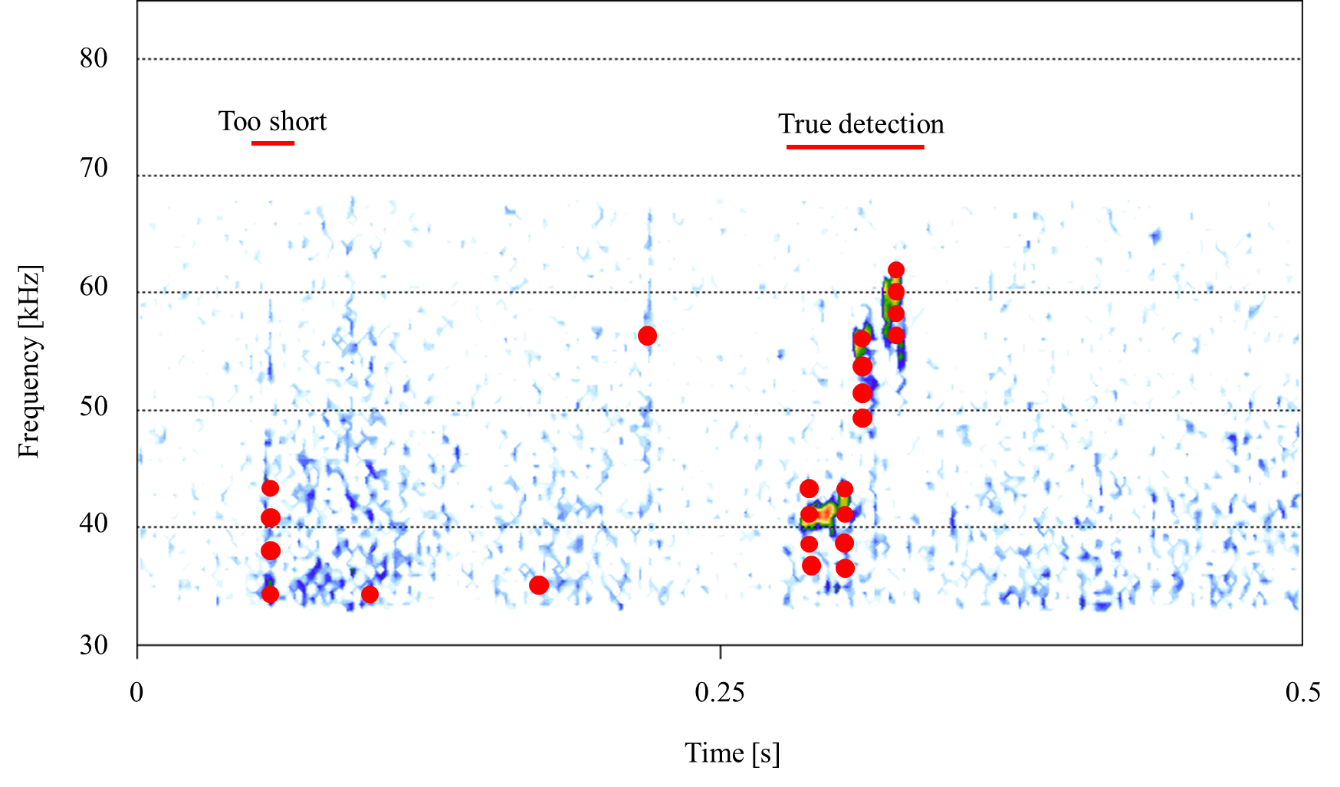


Step 5. Advance the sliding window for 0.5 seconds and repeat aforementioned procedure until reaching the end of the audio.


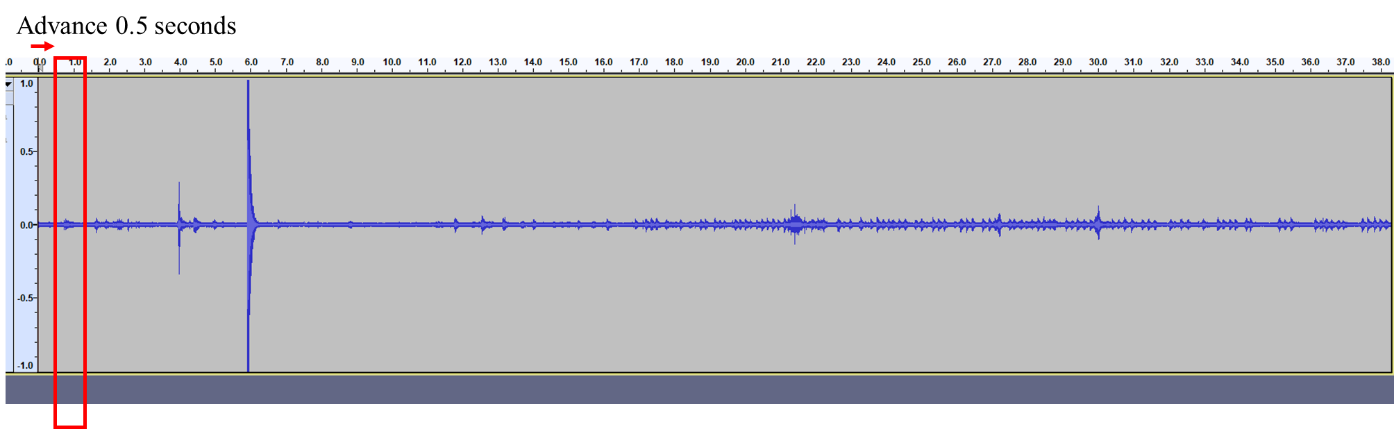


Step 6. Repeat the entire process but place the initial sliding window at 0.25 seconds after the start of the audio recording.


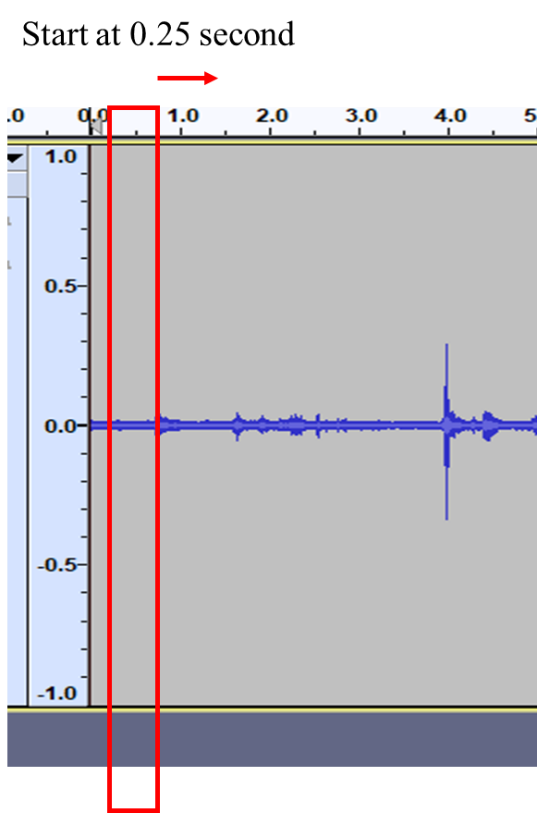


Step 7. Combine detections separated by the edges of the sliding windows.
